# UBA1 inhibition contributes radiosensitization of glioblastoma cells *via* blocking DNA damage repair

**DOI:** 10.3389/fphar.2023.1073929

**Published:** 2023-03-07

**Authors:** Changyong Wu, Yang Shen, Lin Shi, Junhao Zhang, Tongxuan Guo, Lingni Zhou, Wanzhou Wang, Xu Zhang, Rutong Yu, Xuejiao Liu

**Affiliations:** ^1^ Insititute of Nervous System Diseases, Xuzhou Medical University, Xuzhou, Jiangsu, China; ^2^ Department of Neurosurgery, Affiliated Hospital of Xuzhou Medical University, Xuzhou, Jiangsu, China; ^3^ Department of general surgery, The Second Affiliated Hospital of Xuzhou Medical University, Xuzhou, Jiangsu, China

**Keywords:** GBM, TAK-243, radiosensitization, DNA damage repair, cell apoptosis

## Abstract

Glioblastoma multiforme (GBM) is a brain tumor with high mortality and recurrence rate. Radiotherapy and chemotherapy after surgery are the main treatment options available for GBM. However, patients with glioblastoma have a grave prognosis. The major reason is that most GBM patients are resistant to radiotherapy. UBA1 is considered an attractive potential anti-tumor therapeutic target and a key regulator of DNA double-strand break repair and genome replication in human cells. Therefore, we hypothesized that TAK-243, the first-in-class UBA1 inhibitor, might increase GBM sensitivity to radiation. The combined effect of TAK-243 and ionizing radiation on GBM cell proliferation, and colony formation ability was detected using CCK-8, colony formation, and EdU assays. The efficacy of TAK-243 combined with ionizing radiation for GBM was further evaluated *in vivo*, and the mechanism of TAK-243 sensitizing radiotherapy was preliminarily discussed. The results showed that TAK-243, in combination with ionizing radiation, significantly inhibited GBM cell proliferation, colony formation, cell cycle arrest in the G2/M phase, and increased the proportion of apoptosis. In addition, UBA1 inhibition by TAK-243 substantially increased the radiation-induced γ-H2AX expression and impaired the recruitment of the downstream effector molecule 53BP1. Therefore, TAK-243 inhibited the radiation-induced DNA double-strand break repair and thus inhibited the growth of GBM cells. Our results provided a new therapeutic strategy for improving the radiation sensitivity of GBM and laid a theoretical foundation and experimental basis for further clinical trials.

## Introduction

Glioblastoma multiforme (GBM) is the most common malignant primary intracranial tumor associated with a meager clinical cure rate. The average survival rate of patients is only 14–16 months, with a 5-year overall survival rate of less than 10%, posing a challenge to the treatment of patients world-wide (Tang et al., 2021; Zhang et al., 2022a; Zhang et al., 2022b). GBM is mainly in an infiltrative growth pattern and difficult to be completely removed by surgical treatment. Postoperative adjuvant radiotherapy and chemotherapy are still the main treatment methods for GBM. As only a few chemotherapeutic drugs are used to treat GBM, radiotherapy is still the standard therapy for the postoperative treatment of GBM (Zhao et al., 2016). However, most GBM patients are insensitive to radiotherapy, and expanding the radiation range or radiation dosage cannot significantly improve the survival rate of patients after radiotherapy (Ali et al., 2020; Werner et al., 2020). On the contrary, increasing the radiation dose beyond the threshold will increase the risk of a patient’s brain damage. Therefore, screening potential targeted drugs and improving the radiosensitivity of GBM are prerequisites in treating GBM and improving the overall efficacy of GBM.

Radiation therapy is a curative treatment for many malignancies and is usually combined with other therapies, such as surgery, chemotherapy, immunotherapy, and traditional therapy (Wang and Tepper, 2021; Zhang and Xiao, 2021; Saidian et al., 2022). Radiotherapy resistance refers to the tumor gradually adapting to the corresponding physical and chemical environment changes in the course of radiotherapy to resist the killing effect of radiation, which greatly limits the clinical application of radiotherapy (Wang et al., 2020; Li R. Q. et al., 2022). Many studies have revealed the cellular response to radiation is a complex process of multi-genes, multi-factors and multi-mechanisms (Ali et al., 2020; Liu Y. P. et al., 2021), but the mechanism of radiotherapy resistance is still not fully elucidated. DNA damage response pathway plays an important role in tumor radiation resistance. It is mainly involved in detecting DNA damage, initiating DNA repair, and regulating cell cycle and apoptosis (Huang and Zhou, 2020). Relevant studies have shown that the DNA damage repair mechanism was highly active in tumor cells. Furthermore, proteins such as ATR, ATM, DNA-PKcs, Chk1, and Chk2 participate in DNA damage repair in GBM cells and play important roles in radiation resistance (Huang and Zhou, 2020; Yue et al., 2020; Alemi et al., 2022). Loss of ATM/Chk2/p53 pathway components accelerated GBM formation and increased GBM resistance to radiotherapy (Squatrito et al., 2010). Chk1 inhibition by SAR-020106 blocked the cell cycle in the S phase in GBM and thus increased the radiotherapy sensitivity (Patties et al., 2019). In addition, proteins with dysregulated expression in tumor cells, such as Bcl2, Pim3, PI3K/Akt, etc., could all mediate radiation resistance by affecting DNA damage repair in tumor cells (Chen et al., 2016; Liu J. et al., 2021; Alemi et al., 2022). Therefore, targeted therapy for tumor radiosensitivity-related genes and combined therapy with radiotherapy may be an effective measures to treat GBM.

The ubiquitin-proteasome system (UPS) can play a role in the proteolytic function and regulate DNA damage response (Tu et al., 2012). Studies found that ubiquitin-activating enzyme UBA1, a key component of the UPS, is required for responses to ionizing radiation (IR) and replication stress in human cells (Moudry et al., 2012). The siRNA-mediated UBA1 knockdown impaired the formation of both ubiquitin conjugates at the sites of DNA damage and IR-induced foci (IRIF) by the downstream components of the DNA double-strand breaks (DSBs) response pathway, 53BP1 and BRCA1. Moreover, PYR-4, the chemical inhibitor of UBA1, prevented the formation of IRIF and severely impaired DSB repair and formation of 53BP1 foci in G1, a marker of response to replication stress (Moudry et al., 2012). Previous studies indicated that UBA1 is an essential upstream enzyme for ubiquitination-dependent signaling of both DSBs and replication stress in human cells, which was of great significance in the maintenance of genome integrity and cancer treatment (Barghout et al., 2019). Our previous research found that TAK-243, the first-in-class inhibitor of UBA1, showed significant antitumor activity against GBM (Zhang et al., 2022b). This led to the hypothesis that TAK-243 could prevent the GBM resistance to radiation by impeding DNA damage repair.

Whether TAK-243 could promote the sensitivity of GBM to radiation is still unclear. This study tested the hypothesis by measuring the effect of TAK-243 on the growth of GBM xenografts in nude mice exposed to radiation. The findings revealed that TAK-243 promoted the GBM sensitivity to radiation by inhibiting DNA damage repair, thus, inhibiting GBM growth.

## Materials and methods

### Cell lines, antibodies and inhibitor

Human normal astrocyte cell line (HA 1800) and human GBM cell lines (LN229 and U251) were cultured in DMEM supplemented with 10% FBS solution at 37°C in 5% CO_2_. The GSC cell line was cultured in Neurobasal™ Medium containing basic fibroblast growth factor, EGF, B27 supplement, heparin, L-glutamine, and N2 supplement to form a GSC-rich neurosphere culture. Cleaved caspase-3 (#9661, 1:1,000), 53BP1 (#4937, 1:200), γ-H2AX (#9718, 1:500 for WB, 1:200 for IF and 1:100 for IHC), Rad51 (#8875, 1:500) and β-actin (#8457, 1:1,000) primary antibodies were purchased from Cell Signaling Technology (CST, MA, United States). TAK-243 from CSNpharm (CSNpharm, Chicago, IL, United States) were dissolved in DMSO to create a 10 mmol/L solution, which was diluted to different concentrations in DMEM medium before use.

### Cell viability assay

LN229 or U251 cells were seeded on 96-well plates with 3,000 cells per well, and each group was repeated with three duplicate wells. After the cells adhered to the well, different concentrations of TAK-243 were added. 10 μL CCK-8 reagent (Vicmed, Jiangsu, China) was added to each well after culturing for 24 h. Incubation was extended for 1 h. Finally, the absorbance at 450 nm wavelength was detected using a microplate reader.

### Colony formation assay

LN229 or U251 cells were seeded in 6-well cell culture plates at 800 cells/well. The cells were incubated overnight, allowing adherence of cells to the plate, followed by the addition of different TAK-243 concentrations, and 0.1% DMSO was added to the control wells. X-ray radiation was delivered at a dose of 0–6 Gy. After treatment with TAK-243 for 24 h, the cells were cultured in the medium without drugs for 14 days. The cells were washed with PBS and fixed with methanol for 30 min, and then were stained with 0.1% crystal violet for 30–60 min. Cell colonies were observed, photographed, and counted.

### GSC proliferation and tumorsphere formation assay

GSC2 cells were seeded into 24-well plates in triplicate at a density of 10,000 cells per well and treated with 0.1% DMSO, 10 nM TAK-243, IR (2 Gy) or combined treatment. Cells were harvested and counted for each treatment group on days 0, 3, 6, 9, and 12.

For GSC2 tumorsphere formation assay, cells treated with increasing doses of IR (0–4 Gy) were plated in 96-well plates at a density of 1,000 cells per well. DMSO or TAK-243 (10 or 20 nM) was added to the GSC medium, respectively. After 10 days, all tumorspheres in each well (*n* = 3) were assessed *via* bright-field microscopy, and then the number of tumorspheres were counted for radiation dose–response analysis.

### EdU incorporation assay

Cell proliferation was detected using the Cell-Light™ EdU Cell Proliferation Detection Kit according to previously published studies (Liu et al., 2019). LN229 or U251 cells were seeded on 96-well plates. After cell adhesion, cells were treated with TAK-243 combined with IR (0 Gy or 4 Gy) for 12 h or 24 h, followed by incubation with 50 μΜ Edu for 2 h. The cells were fixed with 4% paraformaldehyde for 30 min, washed with PBS, and treated with 0.5% Triton X-100 for 10 min. Cells were incubated with 1× Apollo® reaction cocktail for 30 min and stained with DAPI for 15 min. After the cells were washed three times with PBS, photographs were taken under a fluorescence inverted microscope.

### Cell cycle and apoptosis assay

The effects of TAK-243 and/or radiation on the cell cycle distribution and apoptosis of GBM cells were detected by flow cytometry. LN229 or U251 cells were seeded on 6-cm culture dishes, and a certain TAK-243 concentration was added after cell adhesion. IR was performed after 1 h with radiation doses of 0 Gy and 4 Gy, respectively. Cells were harvested after 24 h. For cell cycle assay, the cells were fixed with 70% ice-cold ethanol overnight, washed twice with PBS, and stained with a solution containing propidium iodide (PI)/RNase for 15 min. For cell apoptosis assay, cells were washed twice with cold PBS and stained with Annexin V-FITC using apoptosis detection kit (Kaiji, Jiangsu, China). The cell cycle distribution and apoptosis were detected by flow cytometry and analyzed by flow cytometry software.

### Caspase-Glo 3/7 activity assay

The cells were seeded on 96-well plates and treated with TAK-243 at different concentrations for 1 h. X-ray radiation was performed with doses of 0 Gy and 4 Gy, respectively. The cells were collected after 24 h, and Caspase-Glo 3/7 enzymatic activities were measured according to the manufacturer’s protocol (Promega). Cells treated with TAK-243 alone or combined with IR have been added with 100 μL Caspase-Glo 3/7 reagent and mixed evenly. After 30 min, 200 μL solution was transferred into white-walled multiwell luminometer plates. The luminescence of each group of samples was detected by using GloMax Luminometer.

### Western blotting

LN229 or U251 cells were treated with TAK-243 for 4 h, and then followed by radiation with doses of 0 Gy and 4 Gy, respectively. The total protein was collected after different time for immunoblot analysis as previously described (Zhang et al., 2022b). The protein expression levels of cleaved caspase-3, γ-H2AX and Rad51 were measured by specific antibodies with β-actin as the loading control.

### Immunofluorescence

Immunofluorescence was performed for LN229 or U251 cells and the staining process was as previously described (Tang et al., 2022). Cells were seeded in 24-well plates and treated with TAK-243 for 4 h. And then cells were treated with radiation for 1 h or 12 h. The harvested cells were fixed with 4% PFA for 30 min, blocked with PBS containing 1% BSA for 2 h, and permeabilized with PBS containing 0.3% Triton X-100 for 30 min at room temperature. Next, the cells were incubated with primary antibodies against γ-H2AX and 53BP1 overnight, washed with PBS thrice on the next day, and incubated with secondary antibodies for 1 h at room temperature. The nuclei were stained with DAPI, and cells were observed and photographed under a confocal microscope.

### Animal experiments

Animal experiments were approved by operations followed the norms of humane care, as previously described (Li M. et al., 2022). LN229 (1×10^6^) or primary GBM cells (5×10^5^) were injected into the brain of nude mice *in situ* with a stereotaxic apparatus. After 5 days, the nude mouse carrying tumor cells were randomly segregated into four groups. The groups were administered with following treatments: the control group was injected with a vehicle, the drug alone group was administered with 10 mg/kg TAK-243, the radiotherapy alone group was irradiated with 10 Gy, and the combined treatment group was administered with 10 mg/kg TAK-243 and irradiated with a dose of 10 Gy. TAK-243 and vehicle were both injected intraperitoneally twice a week. The radiotherapy alone group received conventional radiotherapy constituting 2 Gy every other day for 5 times. After 4 weeks of treatment, mice in each group were randomly sacrificed, and brains were perfused to observe the size of tumors. The remaining seven mice in each group were used for survival analysis. Mice were euthanized upon manifestation of neurological symptoms such as rotational behavior, reduced activity, dome head and so on caused by tumor progression.

### Hematoxylin-eosin (HE) and immunohistochemistry (IHC) staining

The whole brains of mice in the control and treatment groups were fixed in 4% paraformaldehyde overnight. After paraffin embedding, the tissue was cut into 5 μm sections, fixed on glass slides, and dried in an oven. To perform the HE staining assay, sections were dewaxed in xylene, hydrated by graded alcohol, and rinsed with tap water. Sections were stained successively with hematoxylin and eosin for 5 min and dehydrated and sealed with neutral gum. IHC assay was performed as previously described (Zhang et al., 2020). All the images were observed and photographed under a microscope.

### Statistical analysis

Each experiment was repeated more than 3 times independently. The selected chart was one of the results of repeated experiments. The experimental results were statistically analyzed by using the statistical software GraphPad Prism 6.0. Data were represented as mean ± standard deviation. Comparison between two groups was analyzed by unpaired Student’s *t* test. One-way analysis of variance (ANOVA) was used for the comparison more than two groups. Kaplan-Meier method was used for survival analysis. Log-rank Test was used to compare whether there was a difference in survival time between the two groups. *α* = 0.05 was determined as the test level. *p < 0.05 was considered as statistical significance in all results.

## Results

### TAK-243 combined with radiotherapy significantly inhibited colony formation of GBM cells

**FIGURE.1:**
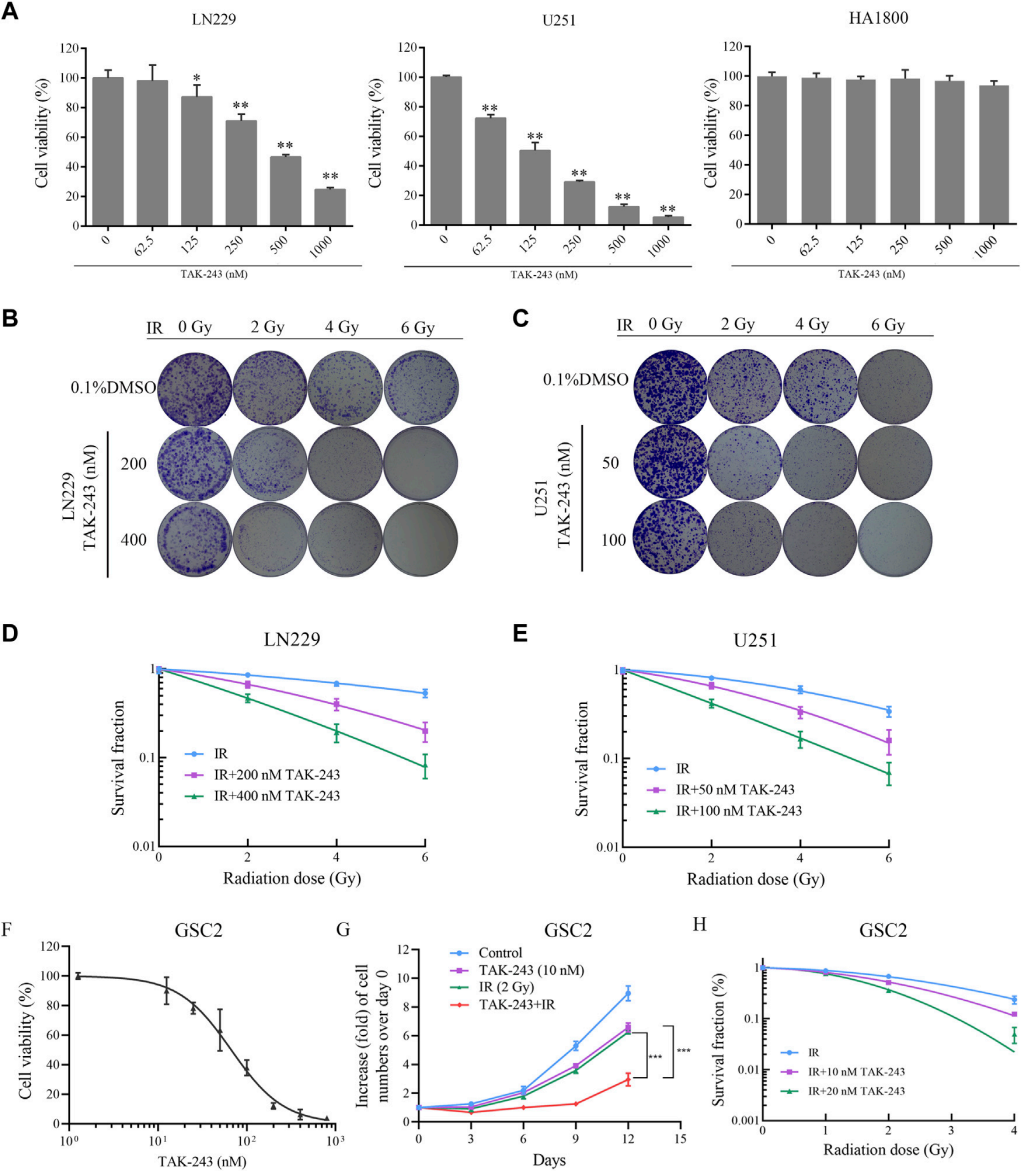
TAK-243 enhances radiation sensitivity of GBM and GSC cells. **(A)** LN229, U251, and HA1800 cells were treated with indicated doses of TAK-243 for 24 h, and cell viability was measured by CCK-8 assay. Representative images **(B, C)** and quantitative results **(D, E)** of colonies of TAK-243-treated LN229 and U251cells after radiation. **(F)** The cell viability of TAK-243-treated GSC2 (0–800 nM) was evaluated by CCK-8 assay. **(G)** GSC2 cells were treated with TAK-243 (10 nM) and/or IR (2 Gy). Cell proliferation rate was evaluated by cell count on indicated days. **(H)** The quantitative results of tumorsphere formation of TAK-243-treated GSC2 cells after IR. The data represent the mean ± SD of three independent experiments, *, *p* < 0.05, **, *p* < 0.01, ***, *p* < 0.001.

To investigate the inhibitory effect of the UBA1 inhibitor TAK-243 on the survival of GBM cells, the CCK-8 experiment was first employed to examine the impact of TAK-243 on the viability of LN229 and U251 cells. The CCK-8 assay findings revealed that TAK-243 significantly inhibited the viability of GBM cells in a dose-dependent manner after 24 h treatment, but no obvious inhibitory effection on HA 1800 (Figure 1A).

To better understand the radiosensitization effect of TAK-243 combined with IR, the colony formation assay was performed to assess the efficiency of clone formation. The data showed a decrease in the colony formation rate of LN229 and U251 cells with an increase in radiation dose. At the same radiation dose, compared with the IR alone group, the colony formation rate of TAK-243 combined with IR decreased significantly (Figures 1B, C). Statistical analysis revealed a dramatic decrease in the survival curve of cells treated with TAK-243 combined with IR compared to the IR group (Figures 1D, E). The above results showed that TAK-243 combined with IR could significantly reduced the colony formation ability of LN229 and U251 cells in a dose-dependent manner.

### TAK-243 increased the sensitivity of GSC cells to radiation

GSC subpopulations has been considered as one of the factors contributing to the radiotherapy resistance in GBM patients after treatment (Galdieri et al., 2021). Inhibiting GSC survival is an effective treatment strategy to improve radiotherapy sensitivity (Chen et al., 2022). In this study, we also assessed the effect of TAK-243 on the viability of GSC cells. We found that TAK-243 significantly inhibited the viability of GSC2 cells in a dose-dependent manner after 72 h treatment (Figure 1F).

Furthermore, we examined the impact of TAK-243 combined with IR on GSC cell proliferation and tumorsphere formation. The results were shown in Figure 1G, compared with the IR alone group or TAK-243 alone group, TAK-243 treatment with IR resulted in a significant decrease in the proliferation rate of GSC2 cells. We also found that TAK-243 treatment markedly sensitized GSC2 cells to IR treatment as determined using tumorsphere formation assays (Figure 1H). Taken together, TAK-243 can increase the sensitivity of GSC cells to radiation.

### TAK-243 combined with IR inhibited cell proliferation and arrested cell cycle in the G2/M phase

**FIGURE.2:**
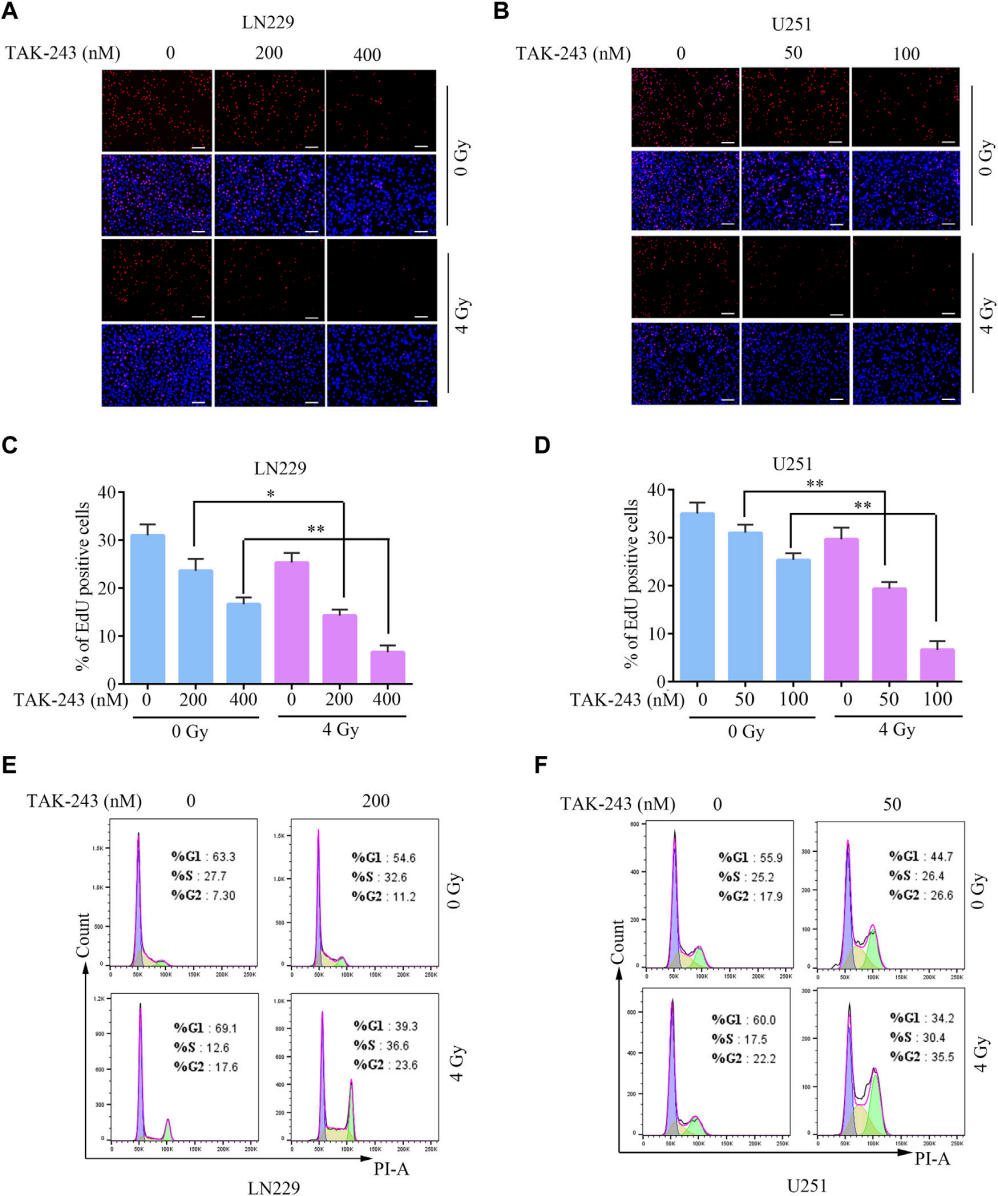
TAK-243 combined with IR inhibits GBM cell proliferation and induces cell cycle arrest. Evaluation of anti-proliferation effects of TAK-243 and/or IR by EdU incorporation assay. **(A, B)** Represent images were showed, scale bar: 100 μm. **(C, D)** The percentage of EDU positive cells was presented as the ratio of EdU positive cells to total DAPI positive cells. All the results were expressed as mean ± SD, *, *p* < 0.05, **, *p* < 0.01. **(E, F)** The cell cycle distribution of GBM cells treated with TAK-243 combined with IR was detected by flow cytometry.

To further elucidate the function of TAK-243 in increasing the radiosensitivity of GBM cells, EdU incorporation assay was employed to determine the proliferation rate of LN229 and U251 cells treated with TAK-243 and/or IR. Compared with control group, the percentage of EdU positive cells of LN229 were decreased by 23.65% and 46.23% on average under 200 and 400 nM TAK-243 treatment, respectively. While on 4 Gy dosage treatment, the percentage of EdU positive cells decreased by 53.77% and 78.48% when treated with 200, and 400 nM TAK-243, respectively. Similar results were also observed in U251 cells. To better study the kinetics of DNA synthesis, we also assessed cell proliferation rate at earlier time points. After treatment for 12 h, we have found that TAK-243 combined with IR inhibited GBM cell proliferation more effectively than the IR alone group or TAK-243 treatment alone (Supplementary Figure S1). Taken together, TAK-243 combined with IR inhibited GBM cell proliferation more effectively than the IR alone group or the drug alone group (Figures 2A–D).

Flow cytometry was used to analyze the potential mechanism of TAK-243 combined with IR in inhibiting cell proliferation, and the impact of TAK-243 combined IR on the cell cycle progression of LN229 and U251 cells were analyzed. The results revealed that TAK-243 combined with IR increased the number of cells in the G2/M phase and arrested the cell cycle in the G2/M phase compared with the drug alone or IR alone groups (Figures 2E, F). The above results indicated that TAK-243 combined with IR could induce cell cycle arrest and significantly inhibit cell proliferation.

### TAK-243 combined with IR increased the activity of caspase-3/7 and induced apoptosis

**FIGURE.3:**
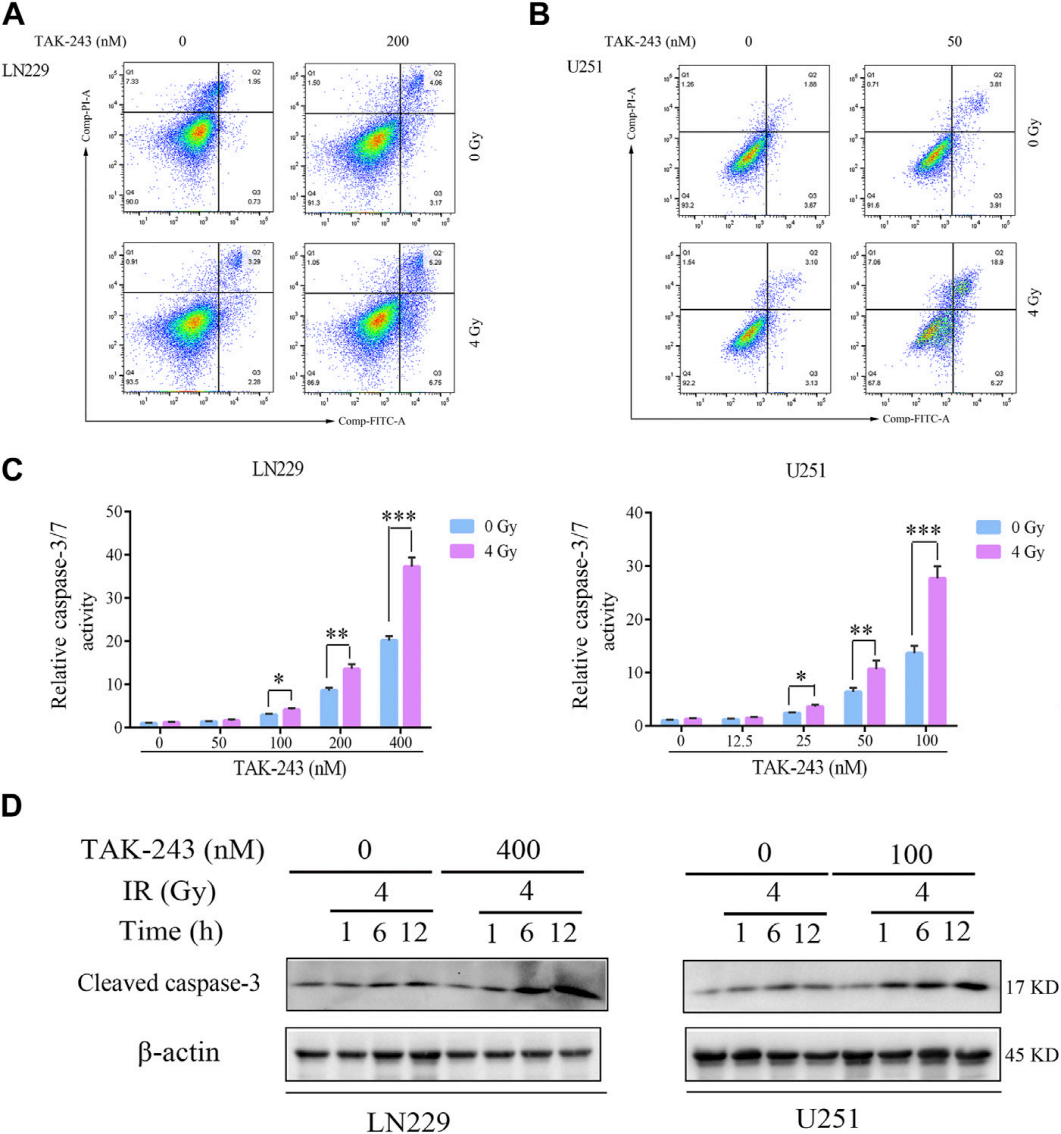
TAK-243 combined with IR enhances GBM cell apoptosis. LN229 **(A)** and U251 **(B)** cells were incubated with TAK-243 and/or IR for 24 h, and then cells were stained with Annexin V/PI. Apoptosis was evaluated by flow cytometry. **(C)** LN229 and U251 cells were treated with TAK-243 (0–400 nM) and/or IR (4 Gy) for 24 h. Caspase 3/7 activity were assessed by Caspase-Glo 3/7 activity assay. **(D)** The protein levels of cleaved caspase-3 were evaluated using immunoblotting in LN229 and U251 cells treated by TAK-243 and/or IR. All the data were presented as means ± SD. *, *p* < 0.05, **, *p* < 0.01.***, *p* < 0.001.

Flow cytometry was performed to analyze the effect of TAK-243 combined with IR on GBM cell apoptosis to further elucidate the effect of TAK-243 and/or IR on the apoptosis of LN229 and U251 cells. Compared with the control group, TAK-243 combined with IR substantially increased the cell apoptotic rate (Figure 3A). Similar results were also obtained in U251 cells (Figure 3B). Compared with the control group, the proportion of apoptosis increased significantly after combined treatment.

Meanwhile, Caspase-Glo® Kit was used to detect the activity of caspase-3/7 of TAK-243 and/or IR-treated LN229 and U251 cells. Compared with the drug alone, TAK-243 combined with IR significantly increased the activity of caspase-3/7 (Figure 3C).

Western blot was used to analyze the effect of TAK-243 combined with radiotherapy treatment on the cleaved caspase-3 levels. Compared with the control group, the amount of cleaved caspase-3 increased in GBM cells treated with TAK-243 combined with radiotherapy (Figure 3D). The results indicated that TAK-243 combined with radiotherapy could increase the activity of caspase-3/7 and then induce the apoptosis of GBM cells.

### TAK-243 combined with IR inhibited DNA double-strand break repair

**FIGURE.4:**
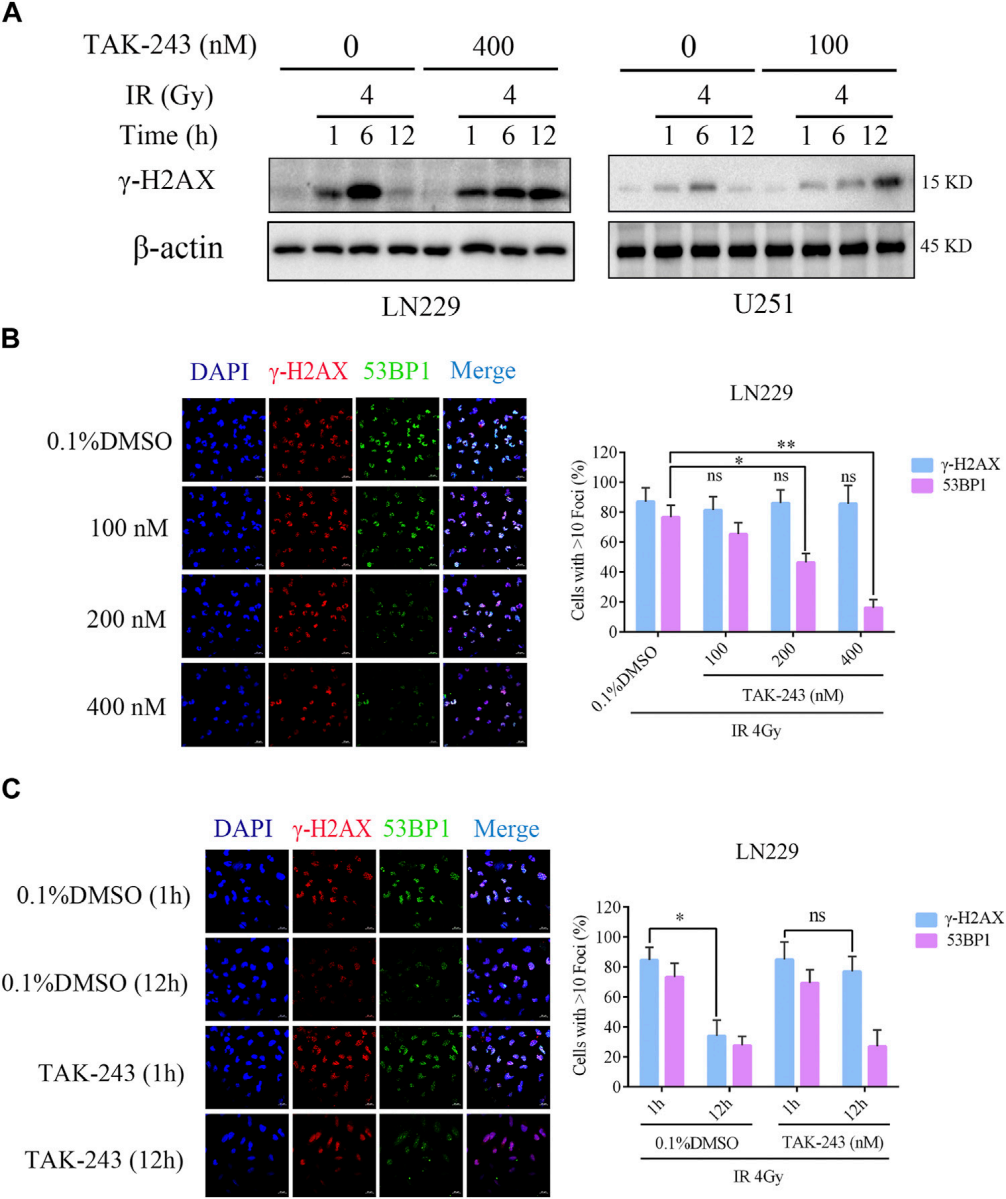
TAK-243 combined with IR inhibits DNA double-strand break repair. **(A)** LN229 and U251 cells were pre-treated with TAK-243 (400 or 100 nM) for 4 h, and followed by IR for indicated time, and then the protein levels of γ-H2AX were assessed by immunoblotting. **(B)** LN229 cells were treated with increasing concentrations of TAK-243 for 4 h, and then followed by IR for 1 h. Cells were examined for subnuclear γ-H2AX and 53BP1 foci by immunofluorescence. Quantitative foci of γ-H2AX and 53BP1 were then analyzed. **(C)** LN229 cells were pretreated with 0.1% DMSO or TAK-243 (100 nM) for 4 h, and combined with IR (4 Gy) for 1 h or 12 h, respectively. Representative images and quantification of γ-H2AX and 53BP1 foci were shown. Values represent the mean ± SD, *, *p* < 0.05, **, *p* < 0.01, ns, non-significant. Scale bar: 20 μm.

To study the effect of TAK-243 on radiotherapy-induced DNA damage repair, the level of DNA damage-related protein, histone H2AX phosphorylation was examined. The results revealed that there was no significant change in histone H2AX phosphorylation level after 4 h of TAK-243 treatment alone without IR. However, with the increase of IR time, the phosphorylation level of H2AX was increased, and the highest expression was found at 6 h. After 12 h, the phosphorylation level of H2AX was decreased, indicating that DNA double strand breaks were gradually repaired over time. However, we found no traces of H2AX phosphorylation level decline when treated with TAK-243 combined with IR over a prolonged period. In U251 cells, we also found that the phosphorylation level of H2AX was dramatically enhanced in the TAK-243 plus IR group, which was consistent with the results in LN229 cells (Figure 4A). We also examined the effect of TAK-243 and/or IR on expression level of Rad51, a crucial DNA double-strand break repair factor. The results showed that Rad51 was increased by IR alone, and TAK-243 treatment attenuated the increased Rad51 level in both LN229 and U251 cells (Supplementary Figure S3).

To further determine the effect of TAK-243 combined with IR on DNA double-strand break repair, we examined IR-induced foci formation of γ-H2AX and 53BP1 after LN229 cells were treated with DMSO or TAK-243. Although all samples showed similar foci formation of γ-H2AX, the recruitment of the downstream effector 53BP1 was impaired in the combined treatment group, with the inhibition of 53BP1 recruitment in a concentration-dependent manner (Figure 4B). Furthermore, LN229 cells were treated with DMSO or 100 nM TAK-243 combined with radiotherapy for 1 h and 12 h, respectively. Compared with radiotherapy alone, treatment with TAK-243 impeded the LN229 cell’s ability to resolve DSBs (γ-H2AX foci) 12 h post-IR. In addition, 12 h post-IR, the TAK-243-treated cells also exhibited reduced foci formation of 53BP1 compared with DMSO-treated controls (Figure 4C). Similary results were obversed in U251 cells (Supplementary Figure S2). The above results preliminarily indicated that TAK-243 could inhibit the repair of DNA double-strand breaks induced by IR, thus increasing DNA damage and inhibiting cell proliferation.

### TAK-243 combined with IR alleviated the growth of GBM tumors *in vivo*

**FIGURE.5:**
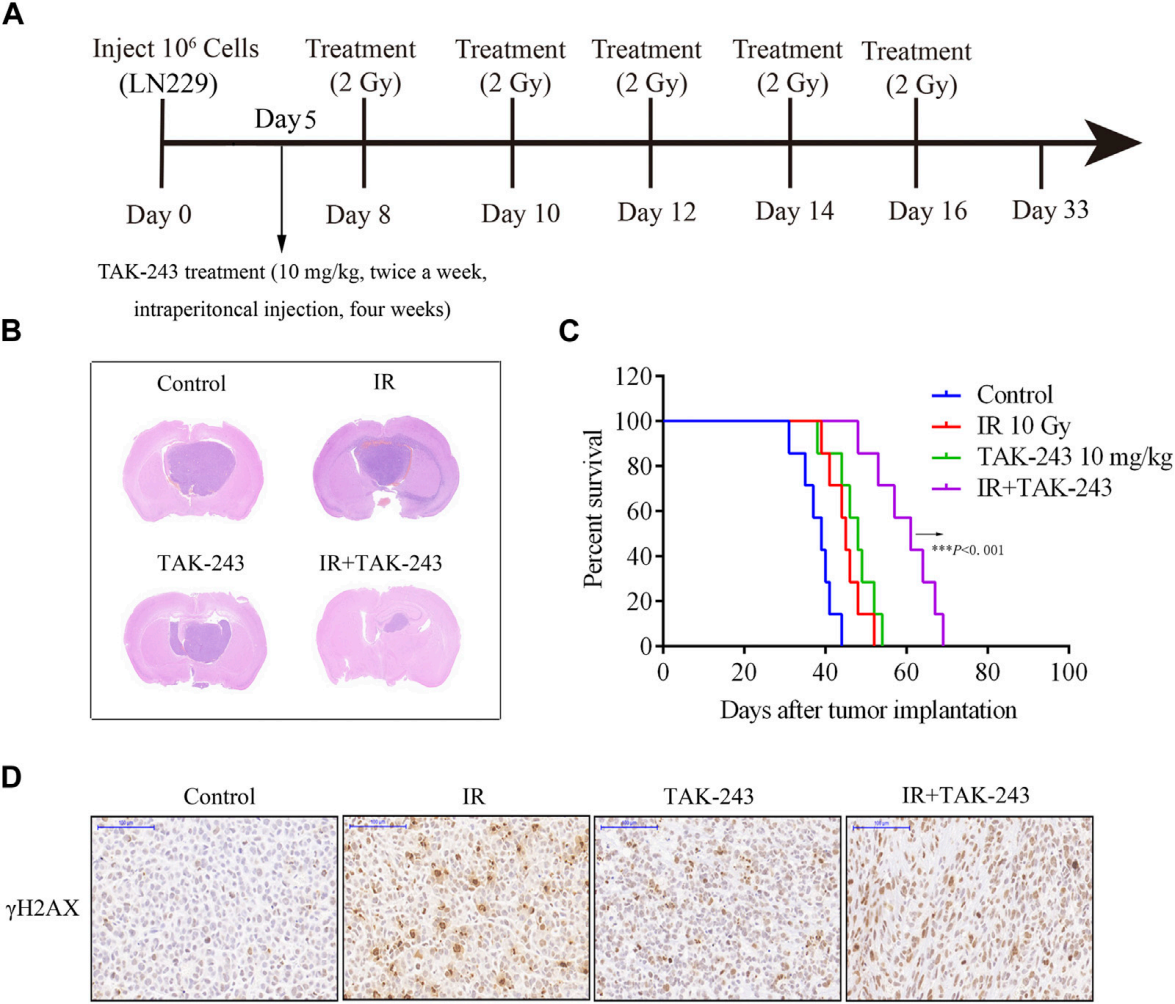
Combination of TAK-243 with IR slows GBM xenograft tumor growth *in vivo* and prolong the survival of tumor-bearing mice. **(A)** Schematic diagram of mice treated with TAK-243 combined with IR. **(B)** Mice bearing LN229-drived xenograft tumor were treated with TAK-243 (10 mg/kg) and/or IR (10 Gy). Representative images of H&E staining of whole brain sections from control group and TAK-243 and/or IR treatment groups. **(C)** The survival analysis of mice with TAK-243 and/or IR treatment, ***, *p* < 0.001. **(D)** The levels of γ-H2AX were examined after TAK-243 and IR co-treatment *in vivo* by IHC assay. Representative images were showed, scale bar: 100 μm.

To further evaluate the therapeutic efficacy of TAK-243 combined with IR in GBM, we constructed a LN229 orthotopic xenograft GBM model in nude mice and performed different treatments as shown in Figure 5A. The findings in the HE staining assay revealed that the tumor volume of the nude mice treated with TAK-243 combined with IR was significantly smaller than that of the control group and TAK-243 alone group, or IR alone group (Figure 5B). The survival analysis of tumor-bearing mice showed that TAK-243 combined with IR could significantly prolong the median survival of tumor-bearing mice (Figure 5C). We further analyze the effect of TAK-243 combined with IR on the expression levels of γ-H2AX *in vivo*. Compared with the control group, TAK-243 alone group or IR alone group, the combination treatment group showed an increased number of γ-H2AX-positive cells, which coincided with the results of the *in vitro* experiments (Figure 5D).

In addition, a primary GBM cell orthotopic xenograft mouse model was also used to assess the efficacy of TAK-243 combined with IR. We found that TAK-243 and IR administration markedly inhibited primary GBM cell growth *in vivo* (Supplementary Figure S4). Taken together, these results indicated that TAK-243 combined with IR could significantly inhibit the growth of intracranial tumor cells *in vivo*.

## Discussion

Radiotherapy is considered as a standard treatment regimen in GBM clinics. However, most GBM patients are resistant to radiotherapy, which is also the main reason for the recurrence of GBM after surgery. Screening effective chemotherapeutic drugs and improving radiotherapy sensitivity are the hotspots in tumor research. In previous studies, we found that TAK-243, the first-in-class UBA1 inhibitor, significantly inhibited GBM cell proliferation and effectively delayed the growth of intracranial GBM in mice. The present study aimed to determine whether TAK-243 could sensitize GBM to radiation therapy. The findings revealed that TAK-243 improved the GBM sensitivity to radiotherapy by inhibiting DNA damage repair response, thus effectively inhibiting GBM cell’s growth.

The ubiquitin-proteasome system (UPS) is the core of cellular proteolysis. UPS utilizes its degradation ability to control and integrate various physiological processes in cells, including signal transduction, DNA damage repair, cell cycle progression, etc, (McBride et al., 2003; Soave et al., 2017). Studies have indicated UPS as a novel therapeutic strategy for cancer treatment, improving patient’s prognosis in other cancer histologies (Ao et al., 2017; Aliabadi et al., 2021). UBA1, the most important E1 enzyme in humans, is responsible for initiating many dysregulated downstream effects in malignant tumors, making it as an attractive target in anticancer strategies (Xu et al., 2013; Barghout et al., 2019; Barghout and Schimmer, 2021; Zhang et al., 2022b). TAK-243 is a first-in-class inhibitor of UBA1 and is currently undergoing multiple phase 1 clinical trials on advanced malignancies (NCT02045095, NCT03816319) (Hwang et al., 2022). Our previous study has shown that TAK-243-induced cell cycle arrest may mainly occur by inducing endoplasmic reticulum stress (Zhang et al., 2022b). However, ionizing radiation induces cell cycle arrest mainly by causing DNA damage (Liu et al., 2020; Sadoughi et al., 2021). So they may have a distinct mechanism. Nevertheless, we also found that G2/M arrest was more significant after TAK-243 combined with IR. The present study clarified that TAK-243 combined with IR could significantly inhibit GBM cell proliferation, arrest the cell cycle in the G2/M phase.

We also found TAK-243 combined with IR significantly induced cell apoptosis. Our previous study showed that TAK-243 enhanced GBM cell apoptosis by inducing endoplasmic reticulum (ER) stress and activating PERK/eIF2/ATF4 and IRE1α/XBP1 signaling pathways, two major unfolded protein response (UPR) activation pathways (Zhang et al., 2022b). Furthermore, activation of UPR increased the activity of caspase family proteins (Abdullah and Ravanan, 2018; Md Nesran et al., 2019). It has been shown that ionizing radiation also induces ER stress and promotes tumor cell apoptosis (Panganiban et al., 2013; Kong et al., 2021). Therefore, we speculated that the increase in apoptosis rate induced by TAK-243 combined with IR might be through over-activation of ER stress and UPR. *In vivo* experiments also further verified that TAK-243 combined with IR effectively inhibited the growth of intracranial tumors in mice and prolonged the survival of tumor-bearing mice. The current results were consistent with the research conclusions in hematological tumors (Barghout et al., 2019; Obiorah et al., 2021) and small cell lung cancer (Majeed et al., 2021), indicating that targeting UBA1 could increase the tumor cell sensitivity to IR, and the combined treatment of TAK-243 and IR may be a promising potential way to cure tumors.

Accurate gene replication and transmission are the basis for maintaining cell balance and biological activity (Mognato et al., 2021). However, external damaging agents and endogenous mutagens destroy DNA integrity and seriously threaten genome stability. If not appropriately repaired, the DNA damage will eventually lead to cancer (Huang and Zhou, 2021). DNA damage response (DDR) is a major agent in the radiation resistance of glioma. This pathway is mainly involved in detecting DNA damage, initiating DNA repair and regulating cell cycle and apoptosis (Ferri et al., 2020; Majd et al., 2021). In vertebrates, DDR is controlled by two major signalling pathways, ATM-Chk2 and ATR-Chk1 protein kinase (Manic et al., 2015). UBA1 is a key regulatory protein for DNA double-strand breaks and genome replication in human cells. Related studies found that UBA1 was recruited to ATR activation structures and bound to ongoing replication forks (Kumbhar et al., 2018). Interference with UBA1 expression or inhibition by PYR41 impedes the phosphorylation of Chk1, thus inactivating the ATR-Chk1 signaling pathway (Kumbhar et al., 2018). In addition, the UBA1 inhibitor, PYR-41, prevented the recruitment of 53BP1 after radiotherapy and inhibited DNA damage repair (Moudry et al., 2012). The present study found that UBA1 inhibition by TAK-243 continuously increased the radiation-induced γ-H2AX expression, while TAK-243 combined with radiotherapy treatment effectively prevented the foci formation of 53BP1 in GBM cells. Our results further confirmed that UBA1 inhibition effectively blocked the repair of DNA double-strand break induced by IR and inhibited GBM cell proliferation.

In conclusion, our results showed that targeting UBA1 inhibited DNA damage repair and enhanced the efficacy of radiotherapy for GBM. The UBA1 inhibitor TAK-243 combined with IR significantly inhibited the colony formation ability of cells *in vitro*, induced cell cycle arrest, and inhibited cell proliferation. The animal experimental results further confirmed that TAK-243 combined with IR effectively prolonged the tumour-bearing mice’s survival rate and inhibited the growth of intracranial tumor cells. Since TAK-243 has been applied in clinical trials, our research provides a theoretical and experimental foundation for further clinical trials of TAK-243 combined with IR in the treatment of tumors.

## Data Availability

The raw data supporting the conclusions of this article will be made available by the authors, without undue reservation.
