# Supplementary material for: UBA1 inhibition contributes radiosensitization of glioblastoma cells via blocking DNA damage repair
Source: Front Pharmacol. 2023 Mar 7;14:1073929. doi: 10.3389/fphar.2023.1073929 (PMC10027716; doi:10.3389/fphar.2023.1073929)
Supplement: Supplementary file 1 [file DataSheet1.docx]

**Supplemental data**


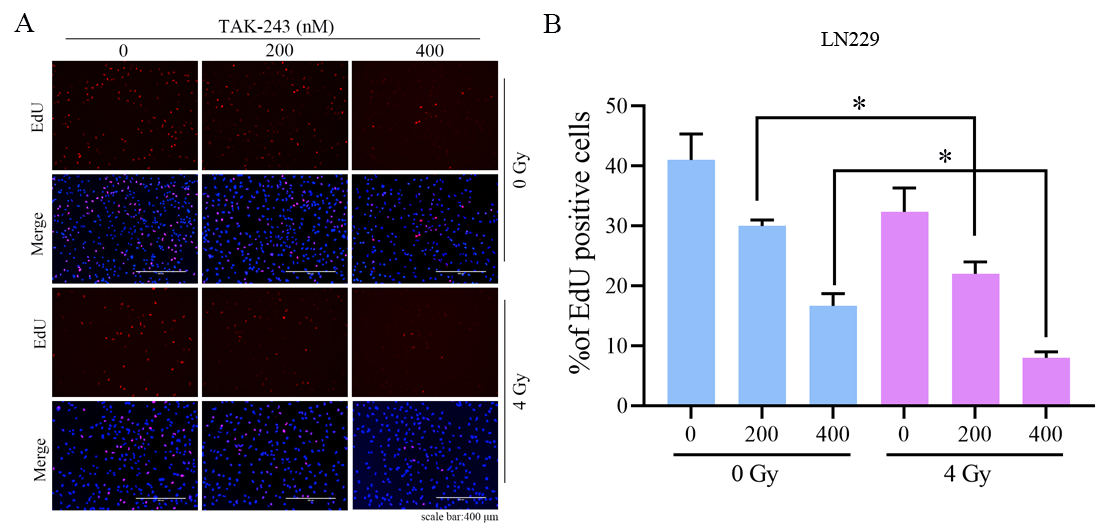


**Figure.S1** Measurement of cell proliferation after treating with TAK-243 or IR alone or their combinations for 12 hours by EdU incorporation assay in LN229 cells. Representative images (A) and statistical analysis (B) for EdU incorporation assay.


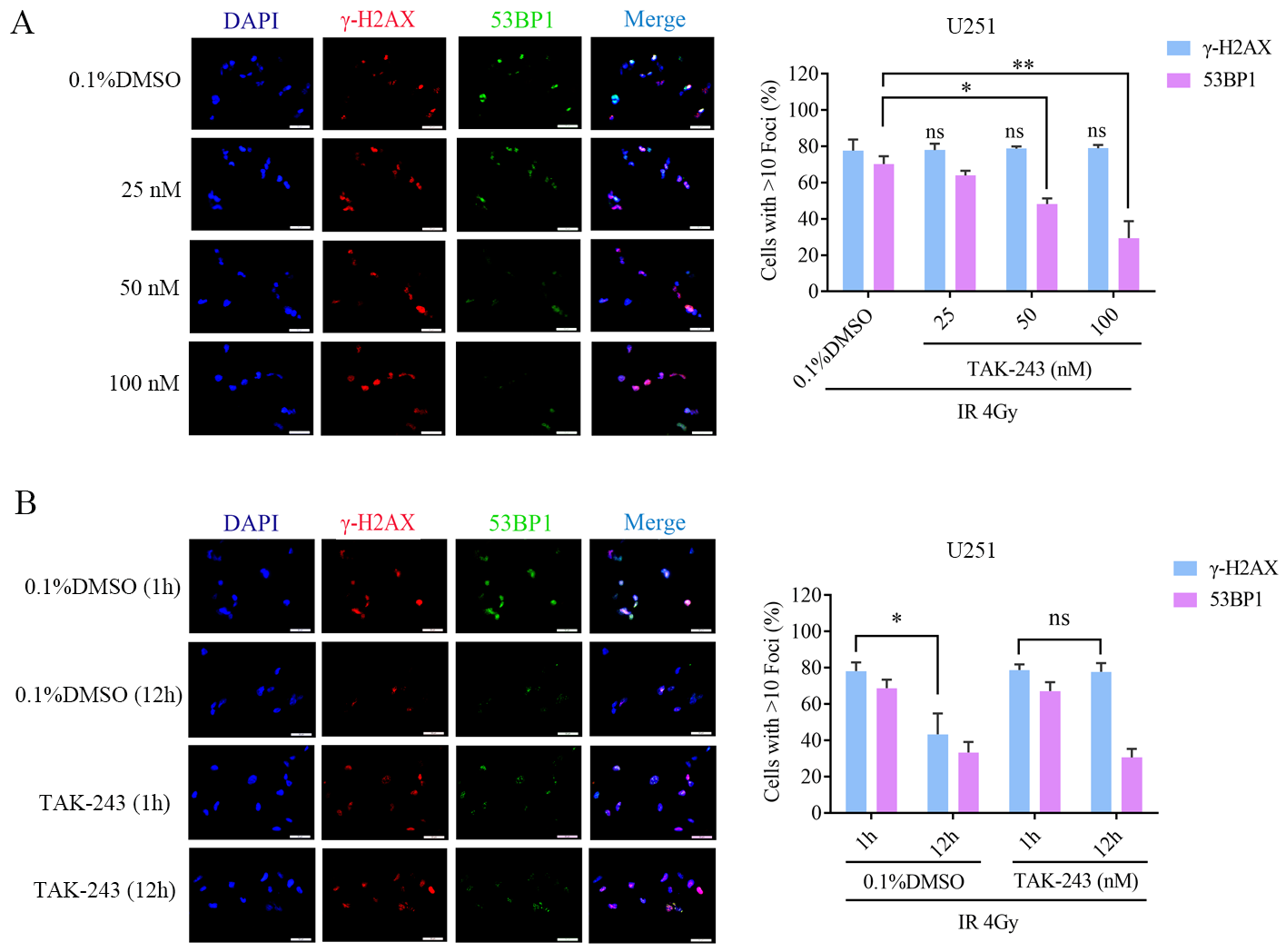


**Figure.S2** Measurement of foci formation of γ-H2AX and 53BP1after treating with TAK-243 combined with IR in U251 cells. (A) U251 cells were treated with increasing concentrations of TAK-243 for 4 hours, and followed by IR (4 Gy) for 1 hour. Cells were examined for subnuclear γ-H2AX and 53BP1 foci by immunofluorescence. Quantitative foci of γ-H2AX and 53BP1 were then analyzed. (B) U251 cells were pretreated with 0.1% DMSO or TAK-243 (25 nM) for 4 hours, and combined with IR (4 Gy) for 1 hour or 12 hours, respectively. Representative images and quantification of γ-H2AX and 53BP1 foci were shown. Values represent the mean ± SD, *, *P*<0.05, **, *P*<0.01, ns, non-significant, scale bar: 50 μm.


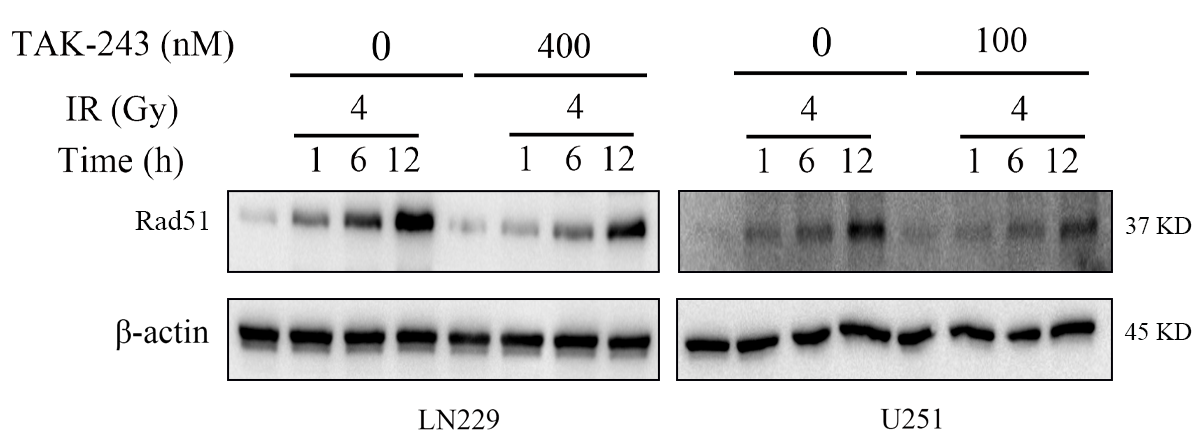


**Figure.S3** Measurement of expression levels of Rad51 after treating with TAK-243 combined with IR in LN229 and U251 cells. LN229 and U251 cells were pre-treated with TAK-243 (400 or 100 nM) for 4 hours, and followed by IR for indicated time. The protein levels of Rad51 were assessed by immunoblotting.


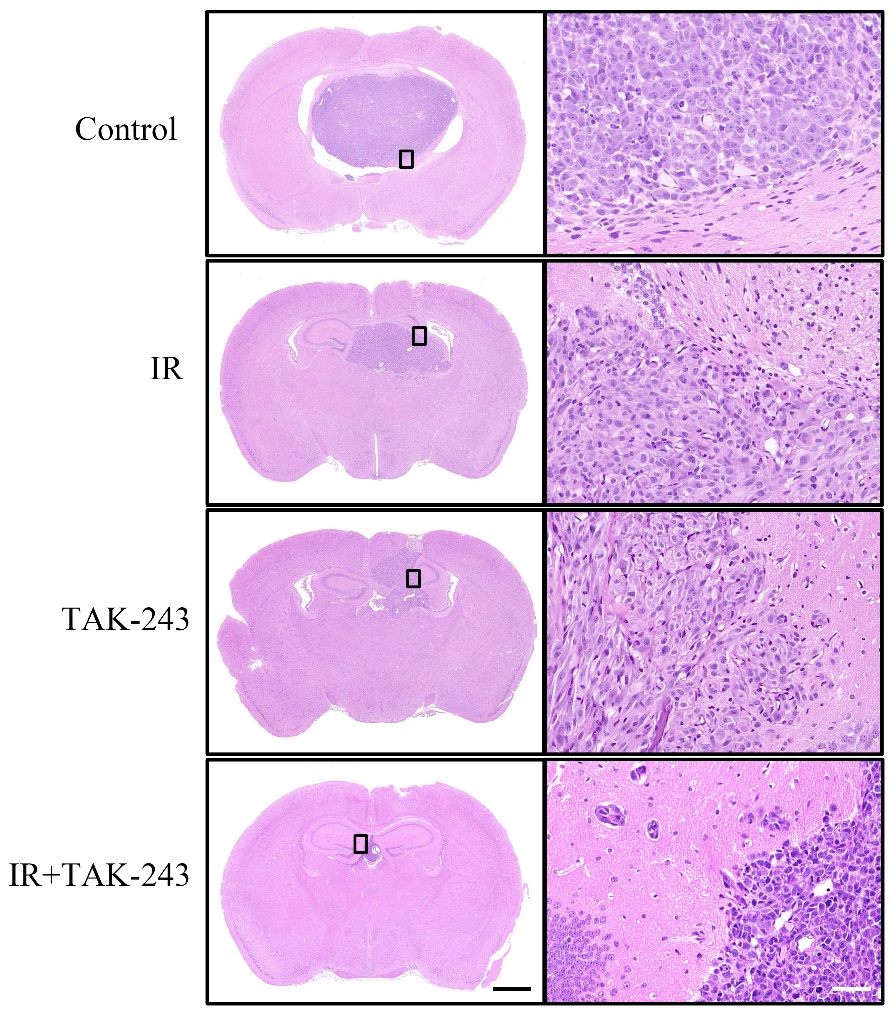


**Figure.S4** Representative images of H&E staining for a primary GBM cell orthotopic xenograft mouse model. Mice were treated with TAK-243 (10 mg/kg) and/or IR (10 Gy). Tumor size was observed by H&E staining, black scale bar: 1000 μm, white scale bar: 50 μm.
